# Paradoxical worsening of hypoxemia in a patient treated by noninvasive positive pressure ventilation for obesity hypoventilation syndrome with concomitant obstructive sleep apnea: a case report

**DOI:** 10.1186/s13256-017-1393-1

**Published:** 2017-08-23

**Authors:** Carole de Picciotto, Coraline Duménil, Olivier Auzel, Violaine Giraud, Marcel Bonay

**Affiliations:** 1 Service de Physiologie-Explorations Fonctionnelles, Hôpital Ambroise Paré, Assistance Publique-Hôpitaux de Paris, Boulogne, France; 2 Service de Pneumologie, Hôpital Ambroise Paré, Assistance Publique-Hôpitaux de Paris, Boulogne, France; 3 Service de Cardiologie, Hôpital Ambroise Paré, Assistance Publique-Hôpitaux de Paris, Boulogne, France; 4 Unité Inserm U1179, laboratoire de physiologie TITAN, UFR des Sciences de la Santé Simone Veil, Université de Versailles St Quentin (UVSQ), Montigny le Bretonneux, France; 5 Hôpital Ambroise Paré, 9 avenue Charles de Gaulle, 92104 Boulogne cedex, France

**Keywords:** Hypoxemia, Obstructive sleep apnea, Hypoventilation, Noninvasive ventilation, Obesity

## Abstract

**Background:**

Noninvasive positive pressure ventilation is frequently prescribed to obese patients with obstructive sleep apnea syndrome and obesity hypoventilation syndrome. However, mechanical ventilation with a positive end-expiratory pressure can induce or worsen a right-to-left shunt through a patent foramen ovale associated with systemic hypoxemia. Thus, in obese patients treated with noninvasive positive pressure ventilation, a paradoxical worsening of hypoxemia may reveal the existence of a patent foramen ovale.

**Case presentation:**

A 50-year-old African woman was referred to our sleep center for severe obstructive sleep apnea syndrome and obesity hypoventilation syndrome. Because she had alveolar hypoventilation and had failed previous obstructive sleep apnea syndrome therapy, noninvasive positive pressure ventilation was started. In May 2015, she had a normal residual apnea/hypopnea index calculated by the ventilator software with no hypoventilation. Six months later, severe hypoxemia without hypercapnia was noted. Contrast transthoracic echocardiography showed right-to-left shunt through a patent foramen ovale. This finding prompted a decrease in expiratory and inspiratory positive airway pressures, after which the ventilator software recorded a normal residual apnea/hypopnea index and the blood gas values improved.

**Conclusion:**

Noninvasive positive pressure ventilation therapy for combined obstructive sleep apnea syndrome and obesity hypoventilation syndrome must be monitored by arterial blood gas measurements, both to reassess the hypercapnia and to look for worsening hypoxemia due to a patent foramen ovale.

## Background

The foramen ovale is a congenital opening between the two cardiac atria that usually closes shortly after birth but may remain patent. Thus, a patent foramen ovale (PFO) is found at autopsy in 20 to 34% of the general population [1]. The PFO represents a channel through which unidirectional blood flow from the right venous blood to the left oxygenated arterial system may occur. The passage of blood from the venous blood into the left arterial blood without lung oxygenation is named right-to-left shunt. It may be associated with hypoxemia, defined in terms of reduced oxygen pressure in the systemic arterial blood (PaO_2_), as detected by an arterial blood gas (ABG) measurement at rest.

Right atrial pressures are elevated in patients with pulmonary hypertension (PH), which may be primary or secondary to another condition such as chronic obstructive pulmonary disease (COPD) or obstructive sleep apnea syndrome (OSAS) [2], and enhance the chance of a right-to-left intracardiac shunt through the foramen ovale. Of 48 patients with OSAS, 33 (69%) had a PFO detectable by contrast transesophageal echocardiography (TEE), compared to only 4 (17%) of 24 controls (*P* < 0.0001) [3]. Systolic pulmonary artery pressure (sPAP) was significantly higher in the patients than in the controls. In the absence of right atrial pressure elevation, right-to-left shunt through the foramen ovale may occur intermittently, sometimes in relation to body position (platypnea-orthodeoxia syndrome). Right atrial pressures may rise abnormally in the absence of PH, for instance during mechanical ventilation with a positive end-expiratory pressure (PEEP) of at least 10 cm H_2_O. In this situation, right-to-left shunt through the PFO worsens the systemic hypoxemia [4–6]. Furthermore, a peak inspiratory airway pressure of 20 cm H_2_O has been shown to improve the TEE detection of PFO [7]. Finally, in a patient with tetralogy of Fallot and severe kyphoscoliosis, noninvasive ventilation paradoxically worsened the hypoxemia by increasing the right-to-left interventricular and right ventriculoatrial pressure gradients [8]. We are not aware of previous reports of paradoxical worsening of hypoxemia due to a right-to-left shunt through a PFO in a patient chronically treated by noninvasive positive pressure ventilation (NPPV) for obesity hypoventilation syndrome (OHS) with concomitant OSAS.

## Case presentation

A 50-year-old African woman was referred to our sleep center for a combination of severe OSAS and OHS. She had a history of systemic hypertension treated since 2010, chronic systemic lupus erythematosus with cutaneous and joint manifestations, a positive serum anticardiolipin antibody test, and Sjögren’s syndrome with lymphocytic interstitial lung disease diagnosed in 2009 and treated by an immunosuppressant and glucocorticoid until 2011.

**Fig. 1:**
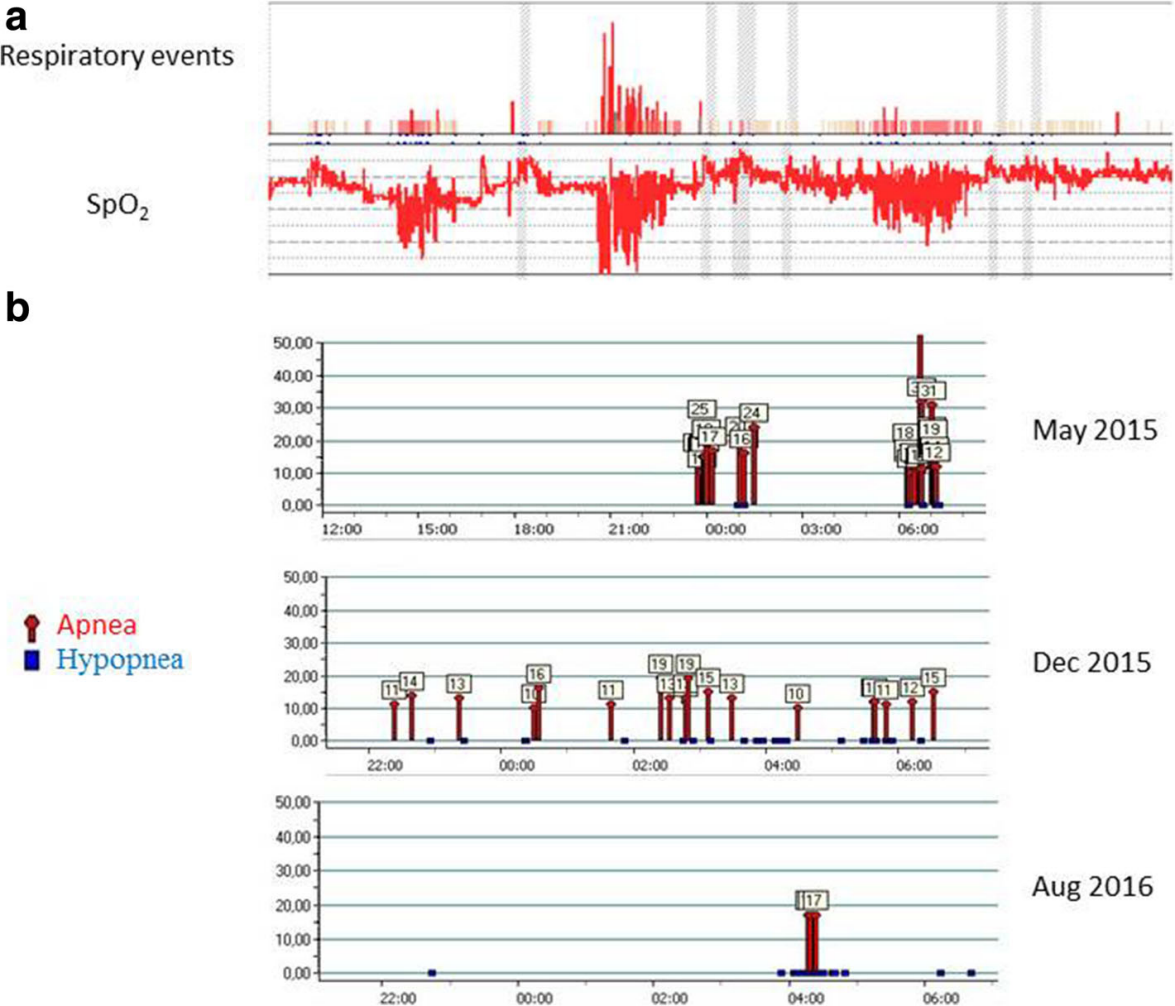
**a** Respiratory polygraphy. *Top*: Obstructive apneas are shown by *solid red bars*, central apneas by *solid gray bars*, obstructive hypopneas by *red speckled bars*, and indeterminate hypopneas by *orange speckled bars*. The height of the bars reflects the duration of the respiratory event. *Bottom*: Oxygen saturation by pulse oximetry. **b** Data from the ResMed ventilator software ResScan: obstructive apneas are in *red* and obstructive hypopneas in *blue*; the numbers above the lines are event durations in seconds. SpO_2_ oxygen saturation by pulse oximetry

Her body mass index was 35.6 kg/m^2^. Consequently, in January 2015, she underwent respiratory polygraphy (CID 102 L; Cidelec, Angers, France) to look for OSAS. Her apnea/hypopnea index (AHI) was 37/hour, indicating severe OSAS. Obstructive hypopneas predominated (Fig. 1a). Total lung capacity and forced expiratory volume in one second/vital capacity (FEV1/VC) were normal but single-breath diffusing capacity of the lungs for carbon monoxide (DLCO) was slightly decreased. ABG analysis evidenced hypoxemia and chronic hypercapnia: PaO_2_, 78 mmHg; carbon dioxide pressure in the systemic arterial blood (PaCO_2_), 48 mmHg; pH, 7.39; calculated bicarbonate level, 29 mmoL/L; and hemoglobin oxygen saturation, 94%. The combination of obesity and alveolar hypoventilation in the absence of lung or neuromuscular disease established the diagnosis of OHS.

She had previously discontinued continuous positive airway pressure (CPAP) therapy due to discomfort with a feeling of suffocation. Given the alveolar hypoventilation, she was therefore started on NPPV (S9 VPAP; ResMed Corp., San Diego, CA, USA) in spontaneous timed (ST) mode, with an expiratory positive airway pressure (EPAP) of 7 cm H_2_O, an inspiratory positive airway pressure (IPAP) of 16 cm H_2_O, and a pressure support of 9 cm H_2_O. In May 2015, the built-in ventilator software (ResScan) reported an AHI of 5.7/hour. Duration of ventilator use was 5 hours 18 minutes. During nocturnal NPPV, mean transcutaneous carbon dioxide (PtcCO_2_) was 45 mmHg and mean oxygen saturation was 95%, indicating resolution of the hypoventilation. Diurnal ABG analysis confirmed this finding (PaO_2_, 87 mmHg; PaCO_2_, 43 mmHg; and pH, 7.45).

Polysomnography (Medatec; Ablis, France) during NPPV was performed in December 2015 because our patient reported sleeping poorly. The results showed excellent upper airway patency (AHI, 1/hour); periodic limb movements with an index of 16.9/hour, resulting in sleep fragmentation with a microarousal/arousal index of 15/hour; and a mean oxygen saturation of 92.2%.

**Fig. 2:**
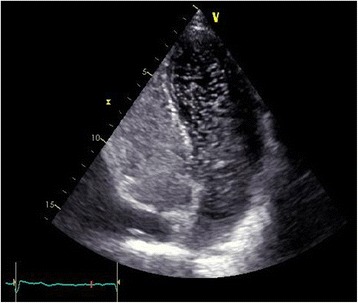
Contrast-enhanced transthoracic echocardiography. Passage of microbubbles to the left atrium during the first three beats after right atrial opacification

In late December 2015, shortly after the polysomnography, she reported worsening exertional dyspnea. ABG analysis was performed to assess this symptom and as part of the routine monitoring of alveolar hypoventilation. Her hypoxemia had worsened (PaO_2_, 53.7 mmHg), whereas PaCO_2_ and pH were normal (42.8 mmHg and 7.43 respectively). She was admitted with transient nasal oxygenotherapy. Computed tomography pulmonary angiography showed dilatation of her pulmonary artery with no evidence of pulmonary embolism. During saline-contrast transthoracic echocardiography with and without Valsalva maneuvers, early passage of over 30 bubbles into the left side of her heart was noted, suggesting PFO (Fig. 2). Her left ventricular ejection fraction was normal and the estimated sPAP was 50 mmHg. Nasal oxygenotherapy was stopped after the diagnosis of PFO and she was monitored in the hospital for a few days, during which her pulse oximetry values improved, suggesting intermittent opening of the foramen ovale.

In January 2016, sPAP value was again estimated at 50 mmHg, confirming the diagnosis of PH, which was ascribed to left-sided heart disease. The finding by computed tomography of smooth septal thickening in a perihilar and gravitational distribution prompted a diuretic challenge. After the treatment, transthoracic echocardiography showed normal sPAP (35 mmHg) and left ventricular ejection fraction, confirming the diagnosis of pulmonary edema.

Given the right-to-left shunt through a PFO, the NPPV parameters were changed in January 2016: the EPAP was decreased to 6 cm H_2_O and the IPAP to 12 cm H_2_O. In August 2016, the ventilator software reported a normal AHI of 3.4/hour (﻿Fig. 1b﻿)﻿ and ABG showed a mild hypercapnia and no hypoxemia (PaO_2_, 85 mmHg and PaCO_2_, 45.7 mmHg). These blood gas values have remained unchanged for several months (Table 1).

**Table 1:** Time-course of arterial blood gas values and expiratory/inspiratory positive airway pressures used for noninvasive positive pressure ventilation

	PaO_2_ mmHg	PaCO_2_ mmHg	Hemoglobin oxygen saturation %	NPPV	Duration of NPPV use	EPAP cm H_2_O	IPAP cm H_2_O	AHI
January 2015	78.4	48	95.5	0	/	/	/	37
May 2015	87	43	97	+	5 hours 18 minutes	7	16	5.7
December 2015	53.7	42.8	90	+	3 hours 30 minutes	7	16	5
August 2016	85	45.7	96.7	+	4 hours 39 minutes	6	12	3.4

*AHI* apnea/hypopnea index, *EPAP* expiratory positive airway pressure, *IPAP* inspiratory positive airway pressure, *NPPV* noninvasive positive pressure ventilation, *PaCO*_*2*_ carbon dioxide pressure in the systemic arterial blood, *PaO*_*2*_ oxygen pressure in the systemic arterial blood

## Discussion

That mechanical ventilation with PEEP can induce or worsen a right-to-left shunt through a PFO is well documented [4, 5]. In contrast, to the best of our knowledge, a single study has assessed the hemodynamic effects of noninvasive ventilation in patients with OHS, and it had only 30 patients [9]. Right ventricular overload was found in 43.3% of patients, that is, slightly above the 20 to 40% prevalence of PH reported in isolated OSAS [10, 11]. After 6 months of NPPV, there was a significant decrease in sPAP [9]. This result requires confirmation in larger studies.

In our patient, OHS and OSAS may have contributed to the PH. The sPAP decline induced by diuretic therapy suggests left-sided heart disease. The mechanism by which PH secondary to left-sided heart disease may have contributed to induce right-to-left shunt through the PFO, leading to a paradoxical aggravation of the hypoxemia, remains unknown. In a patient who had OHS and OSAS with a history of unsuccessful NPPV, closure of the PFO dramatically improved the hypoxemic respiratory failure [12]. In our patient, NPPV probably further increased the right atrial pressure, reversing the pressure gradient between the two atria and inducing right-to-left shunt through the PFO. Furthermore, the PaO_2_ fluctuations during NPPV treatment in our patient were consistent with intermittent opening of the foramen ovale suggesting that the balance of pressures between the right side and the left side of her heart was very unsteady. It would have been interesting to perform sequential bubble tests in increasing EPAP and IPAP values (under adequate monitoring in an intensive care unit; ICU) to provide definite proof of our hypothesis, but we did not carry out this test because of ethical considerations.

## Conclusion

In conclusion, NPPV therapy for combined OSAS and OHS requires ABG monitoring both to evaluate the course of the hypercapnia and to look for paradoxical worsening of the hypoxemia due to right-to-left shunt through a PFO, particularly as very high pressures may be needed to correct the hypoventilation.

## Abbreviations

ABG: Arterial blood gas
AHI: Apnea/hypopnea index
COPD: Chronic obstructive pulmonary disease
CPAP: Continuous positive airway pressure
DLCO: Diffusing capacity of the lungs for carbon monoxide
EPAP: Expiratory positive airway pressure
FEV1/VC: Forced expiratory volume in one second/vital capacity
ICU: Intensive care unit
IPAP: Inspiratory positive airway pressure
NPPV: Noninvasive positive pressure ventilation
OHS: Obesity hypoventilation syndrome
OSAS: Obstructive sleep apnea syndrome
PaCO_2_: Carbon dioxide pressure in the systemic arterial blood
PaO_2_: Oxygen pressure in the systemic arterial blood
PEEP: Positive end-expiratory pressure
PFO: Patent foramen ovale
PH: Pulmonary hypertension
PtcCO_2_: Transcutaneous carbon dioxide
sPAP: Systolic pulmonary artery pressure
ST: Spontaneous timed
TEE: Transesophageal echocardiography
